# Atorvastatin and Conditioned Media from Atorvastatin-Treated Human Hematopoietic Stem/Progenitor-Derived Cells Show Proangiogenic Activity *In Vitro* but Not *In Vivo*

**DOI:** 10.1155/2019/1868170

**Published:** 2019-07-16

**Authors:** Witold N. Nowak, Hevidar Taha, Joanna Markiewicz, Neli Kachamakova-Trojanowska, Jacek Stępniewski, Damian Klóska, Urszula Florczyk-Soluch, Rafał Niżankowski, Marzena Frołow, Zbigniew Walter, Józef Dulak, Alicja Józkowicz

**Affiliations:** ^1^Department of Medical Biotechnology, Faculty of Biochemistry, Biophysics and Biotechnology, Jagiellonian University, Gronostajowa 7, 31-007 Kraków, Poland; ^2^Basic Science Department, College of Agriculture, University of Duhok, Zakho Street 38, 1006 AJ Duhok, Kurdistan Region, Iraq; ^3^Bionanoscience and Biochemistry Laboratory, Malopolska Centre of Biotechnology, Jagiellonian University, 30-387 Krakow, Poland; ^4^International Associated Laboratory, Malopolska Centre for Biotechnology, Jagiellonian University, 30-387 Kraków, Poland; ^5^2^nd^ Department of Internal Medicine, Jagiellonian University Medical College, Skawińska 8, 31-066 Kraków, Poland; ^6^Clinics of Hematology, University Hospital, Jagiellonian University Medical College, M. Kopernika 36, 31-501 Kraków, Poland; ^7^Kardiomed Silesia, M. Curie-Skłodowskiej 10C, 41-800 Zabrze, Poland

## Abstract

Myeloid angiogenic cells (MAC) derive from hematopoietic stem/progenitor cells (HSPCs) that are mobilized from the bone marrow. They home to sites of neovascularization and contribute to angiogenesis by production of paracrine factors. The number and function of proangiogenic cells are impaired in patients with diabetes or cardiovascular diseases. Both conditions can be accompanied by decreased levels of heme oxygenase-1 (HMOX1), cytoprotective, heme-degrading enzyme. Our study is aimed at investigating whether precursors of myeloid angiogenic cells (PACs) treated with known pharmaceuticals would produce media with better proangiogenic activity *in vitro* and if such media can be used to stimulate blood vessel growth *in vivo*. We used G-CSF-mobilized CD34^+^ HSPCs, FACS-sorted from healthy donor peripheral blood mononuclear cells (PBMCs). Sorted cells were predominantly CD133^+^. CD34^+^ cells after six days in culture were stimulated with atorvastatin (AT), acetylsalicylic acid (ASA), sulforaphane (SR), resveratrol (RV), or metformin (Met) for 48 h. Conditioned media from such cells were then used to stimulate human aortic endothelial cells (HAoECs) to enhance tube-like structure formation in a Matrigel assay. The only stimulant that enhanced PAC paracrine angiogenic activity was atorvastatin, which also had ability to stabilize endothelial tubes *in vitro*. On the other hand, the only one that induced heme oxygenase-1 expression was sulforaphane, a known activator of a HMOX1 inducer—NRF2. None of the stimulants changed significantly the levels of 30 cytokines and growth factors tested with the multiplex test. Then, we used atorvastatin-stimulated cells or conditioned media from them in the Matrigel plug *in vivo* angiogenic assay. Neither AT alone in control media nor conditioned media nor AT-stimulated cells affected numbers of endothelial cells in the plug or plug's vascularization. Concluding, high concentrations of atorvastatin stabilize tubes and enhance the paracrine angiogenic activity of human PAC cells *in vitro*. However, the effect was not observed *in vivo*. Therefore, the use of conditioned media from atorvastatin-treated PAC is not a promising therapeutic strategy to enhance angiogenesis.

## 1. Introduction

Development of new blood vessels is necessary for the effective tissue regeneration. Therefore, the mechanisms which allow for the regulation of angiogenesis are of great importance. The process of neovascularization can be influenced not only by the residual endothelial cells but also by the circulating cells. Such myeloid angiogenic cells (MAC) [1] of bone marrow origin were known as endothelial progenitor cells (EPCs). EPCs are enriched in the population coexpressing CD34, CD133, and KDR with a low or absent expression of pan-hematopoietic marker CD45 [1]. In the cell culture, they can bind *Ulex europaeus* lectin and acetylated low-density lipoprotein (LDL) [2].

However, the features initially ascribed to endothelial progenitor cells, *i.e*., phenotype, expression of endothelial markers, and ability to form tube-like structures on Matrigel, can be mimicked by blood monocytes [3]. Cells previously known as endothelial progenitor cells are considered now to be rather myeloid angiogenic cells than endothelial precursors [4]. Endothelial progenitors should be devoid of hematopoietic markers and able to form vessels both *in vitro* and *in vivo*. This change not only is of nomenclature significance but also indicates for the different mechanisms of action, necessary to be investigated for the better translational application [5]. In our study, we worked on cells derived from the CD34^+^ hematopoietic stem and progenitor cells, enriched with CD133^+^ population, and used culture media promoting growth of hematopoietic progenitors. Therefore, the cells we used should be considered as hematopoietic precursors of myeloid angiogenic cells and are referred as PACs. One may speculate that more mature MAC would have better paracrine proangiogenic activity than PAC. However, we decided to use PAC over MAC based on our pilot study, in which media promoting the growth of hematopoietic progenitors gave us the highest yield of cells out of all tested serum-free compositions.

Importantly, conditioned media from MAC better induce endothelial cell migration than those from human umbilical vein endothelial cells (HUVECs) [6]. HUVEC cells treated with the MAC-conditioned media upregulated antioxidant genes coding for catalase, copper/zinc superoxide dismutase, and manganese superoxide dismutase and were more resistant to oxidative stress and apoptosis [7]. Moreover, MAC produce proangiogenic microvesicles, which improve vascularization of pancreatic islets [8] and protect kidneys from ischemia-reperfusion injury [9]. Microvesicles from phenotypical EPC enhance vascularization in ischemic hind limbs in an animal model [10]. Microvesicles derived from phenotypical EPC can also induce angiogenic program in endothelial cells with the transfer of mRNA encoding for transcripts involved in the PI3K/Akt pathway [11]. Media from MAC or EPC potentially could be used for regenerative therapy. Therefore, we aimed to evaluate if active compounds of medicines, or conditioned media from human peripheral blood PAC stimulated with known pharmaceuticals, especially statins, could improve the angiogenesis both *in vitro* and *in vivo*.

Statins are inhibitors of 3-hydroxy-3-methyl-glutaryl-coenzyme A (HMG-CoA) reductase—an enzyme that controls the production of cholesterol. Therefore, statins are a treatment of choice for lowering the cholesterol levels and normalization of the high-density lipoprotein (HDL)/LDL ratio. Apart from their influence on the cholesterol synthesis, statins can also affect the biology of both endothelial cells and circulating proangiogenic cells. Statins enhance mobilization of proangiogenic cells from the bone marrow [12] *via* the PI3K/Akt pathway [13]. The positive effect of statins on the mobilization of proangiogenic cells was observed as well in the diabetic patients with atherosclerotic vascular disease [14], acute coronary syndromes, congestive heart failure, hypertension, hypercholesterolemia, and vasoplastic anaemia (reviewed in [15]). Longer administration of statins, i.e., longer than three months, could lead to the decrease in numbers of circulating proangiogenic cells [16]. Atorvastatin increased the proangiogenic cell proliferation and reduced their senescence [17] and levels of age-related reactive oxygen species [18]. Furthermore, proangiogenic cells pretreated with statins were more resistant to apoptosis caused by oxidized LDLs (oxLDLs) [19] and homocysteine [20]. Stimulation of peripheral blood mononuclear cells with simvastatin enhanced the expression of endothelial markers and production of proangiogenic interleukin (IL) 8 [21], which seemed to be one of the most critical EPC-derived paracrine mitogens for the mature endothelium [22]. Therefore, our objective was to investigate if PAC stimulated with high doses of atorvastatin change the profile of secreted growth factors and immune modulators.

Finally, statins can induce expression of cytoprotective and proangiogenic heme oxygenase-1 via the ERK/p38 MAPK/PKG [23], C/EBP*β*, and AP-1 [24] in murine macrophages. The effect of statins on the heme oxygenase-1 expression in circulating CD34^+^ HSPCs remains so far unknown. Heme oxygenase-1 (encoded by the *HMOX1* gene) is an enzyme degrading heme to carbon monoxide (CO), biliverdin, and Fe^2+^ ions.

Importantly, the polymorphism of the *HMOX1* gene promoter affects the expression of heme oxygenase-1 in humans. Our team has shown that the long promoter alleles (with more GT repeats) result in the lower *HMOX1* expression [25]. Endothelial cells isolated from the people with the long promoter are more sensitive to oxidative stress and show lower proliferation response to VEGF than cells isolated from the people with short, more active, promoter alleles [25].

Apart from its enzymatic activity, heme oxygenase-1 influences cell survival, resistance to the oxidative stress, and angiogenesis [26]. We have recently shown that MAC isolated from the bone marrow of *Hmox1* knockout mice present impaired proliferation, migration, and formation of capillaries [27]. Accordingly, in human and mouse mature endothelial cells, heme oxygenase-1 was necessary for the SDF-1-induced migration [28].

Therefore, our last aim was to elucidate the relation between atorvastatin and heme oxygenase-1 in guiding paracrine activity of human PAC.

Circulating proangiogenic cells, since their discovery by Asahara et al. in 1997 [2], have been an attractive subject of many research and clinical trials. Importantly, such cells can be sources of growth factors and immune modulations, which can altogether improve the tissue regeneration. The strategy based on secretome would be attractive for regenerative medicine, since it would not depend on histocompatibility of cells but only on levels of factors they produce. Therefore, this study is aimed at assessing both *in vitro* and *in vivo* whether human PAC can be stimulated with atorvastatin to produce conditioned media with enhanced proangiogenic activity.

## 2. Materials and Methods

### 2.1. Isolation of Human Peripheral Blood CD34^+^

Human peripheral blood CD34^+^ cells were isolated from peripheral blood mononuclear cells (PBMCs) obtained from a healthy volunteer, who was treated for five days with G-CSF (2 injections of 480 *μ*g of G-CSF per day). The research complied with the Declaration of Helsinki and was approved by the local ethics committee. The donor provided written informed consent for the study. PBMCs were obtained with apheresis using a COBE® Spectra Apheresis System in the Clinics of Hematology, Jagiellonian University Medical College, Kraków, Poland. Remaining erythrocytes were removed by centrifugation on the Ficoll Plus gradient (17-1440-02, GE Healthcare). PBMCs were then aliquoted and frozen in either CryoStor CS10 freezing medium (#07930, Stem Cell Technologies) or PBS with 50% FBS and 10% DMSO (DMS666, BioShop).

**Figure 1:**
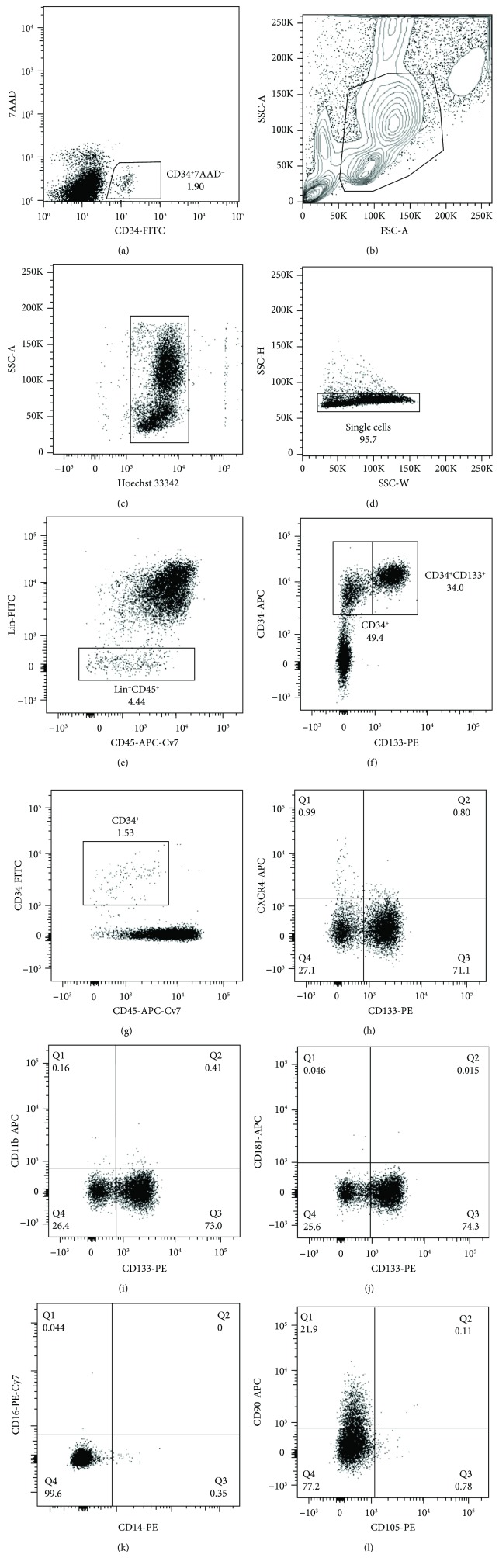
Gating for sorting of CD34^+^7AAD^−^ cells. CD34^+^7AAD^−^ cells constituted 1.6-2% of cryostored PBMCs (a); CD34^+^ cells constituted 1.53% of fresh isolated PBMCs. In majority, they were positive for CD133 (68.8%) and negative for most of tested markers: CD11b, CD14, CD16, CXCR4, and CD105, and 21.9% of them were positive for CD90. Phenotyping of obtained PBMCs was assessed with flow cytometry. Cells (b), Hoechst^+^ nucleated cells (c), singlets (d), Lin^−^ cells (e), CD34^+^ or CD34^+^CD133^+^ cells within Lin^−^Hoe^+^ cells (f), CD34^+^ cells within nucleated cells (g), CXCR4 vs. CD133 within CD34^+^ cells (h), CD11b vs. CD133 within CD34^+^ cells (i), CD181 vs. CD133 within CD34^+^ cells (j), CD16 vs. CD14 within CD34^+^ cells (k), and CD90 vs. CD105 within CD34^+^ cells (l).

When necessary, aliquots of PBMC were thawed, and cells were washed with PBS and treated for 30 minutes with 25 *μ*g/mL DNase I (#10104159001, Roche) in PBS (#21-030-CVR, Corning) with 5.0 *μ*mol/L Mg^2+^ in order to prevent cell clumping. Then, the cells were stained for 25 minutes with an anti-human CD34-FITC antibody (1 : 25, #555821, clone 581, BD Pharmingen) and 10 minutes with 2 *μ*g/mL 7-AAD (#559925, BD Pharmingen) at 4°C. Stained cells were washed with 3 mL of PBS, resuspended in the AutoMACS Running Buffer (#130-091-221, Miltenyi Biotec), and filtered with a 40 *μ*m cell strainer (CLS431750-50EA, Corning). CD34^+^7AAD^−^ cells were sorted on a Beckman Coulter MoFlo XDP FACS sorter (Figure 1(a)).

### 2.2. Cell Culture

Cells were cultured in U-shaped, nonadherent 96-well plates (150 *μ*L, 5.0 × 10^3^ per well) in StemSpan-ACF medium (#09855, Stem Cell Technologies) supplemented with 20% BIT9500 serum replacement (#9500, Stem Cell Technologies), CC100 (#02690, Stem Cell Technologies) mix of cytokines, 50 ng/mL VEGF (#AF-100-20, PeproTech), and penicillin with streptomycin (P4333-100ML, Sigma Aldrich). Compositions of other tested media are shown in Supplementary Table 1. After 48 hours, 50 *μ*L of fresh medium was added; then, 50 *μ*L of medium was changed every other day. On day 6 of culture, cells were stimulated with 7.5, 15, or 30 *μ*mol/L atorvastatin (#3776, Tocris), 7.5, 15, or 30 *μ*mol/L resveratrol (R5010-100MG, Sigma Aldrich), 125, 250, or 500 *μ*mol/L acetylsalicylic acid (A5376-100G, Sigma Aldrich), 2.5, 5.0, or 10 *μ*mol/L sulforaphane (S441-5MG, Sigma Aldrich), or 1.25, 2.5, or 5.0 mmol/L metformin (#317240-5MG, Sigma Aldrich). Conditioned media were collected after 48 hours. The controls, nonconditioned media, contained the same concentrations of stimulants and were kept for 48 hours in a cell culture incubator in the same conditions as stimulated cells. Wash-out conditioned media were collected from stimulated cells after washing with PBS and reseeding on 96-well plates in the complete fresh medium. DMSO, which was used to dissolve all stimulants, was added to the control wells.

### 2.3. Phenotyping of Human Peripheral Blood Mononuclear Cells

Human peripheral blood mononuclear cells from the G-CSF-treated healthy volunteer were stained in an AutoMACS Running Buffer in 4°C with the following antibodies: cocktail of antibodies for hematopoietic lineage markers (Lin) (anti-CD2-FITC (#555326, clone RPA2.10), anti-CD3-FITC (#555332, clone UCHT1), anti-CD14-FITC (#345784, clone M5E2), anti-CD16-FITC (#555406, clone 3G8), anti-CD19-FITC (#555412, clone HIB19), anti-CD24-FITC (#555427, clone ML5), anti-CD56-FITC (#345811, clone NCAM16.2), anti-CD66b-FITC (#555724, clone G10F5), and anti-CD235a-FITC (#559943, clone GA-R2(HIR2), all 1 : 200) all from BD Pharmingen), anti-CD45-APC-Cy7 (#304014, clone 2D1, BD Biosciences, 1 : 50), anti-CD34-APC or anti-CD34-FITC (#555824, #555821, clone 581, BD Pharmingen, 1 : 50), anti-CD133-PE (#130-080-801, clone AC133, Miltenyi Biotec, 1 : 50), anti-CD105-PE (#323206, clone 43A3, BioLegend, 1 : 50), anti-CD90-APC (#559869, clone 5E10, BioLegend), anti-CD181-APC (#551080, clone 5A12, BD Pharmingen), anti-CD184(CXCR4)-APC (#555976, clone 12G5, BioLegend, 1 : 50), anti-CD11b-APC (#101211, clone M1/70, BioLegend, 1 : 50), and 10 *μ*g/mL Hoechst 33342 (B2261-25MG, Sigma Aldrich). Stained cells were then analyzed on a BD LSR II flow cytometer using BD FACS Diva software (Becton Dickinson). Controls for gating are shown in Supplementary Figure 1.

### 2.4. Matrigel Assay

The assay was performed in 96-well plates with 50 *μ*L of growth factor reduced Matrigel (#356231, Corning) per well. 1.0 × 10^4^ HAoEC cells were seeded in each well in 50 *μ*L of EBM-2 medium (#00190860, Lonza) supplemented with 2% FBS. After 30 minutes, 50 *μ*L of conditioned medium was added to the cells. Nonconditioned media with the same stimulants were used as controls. EBM-2 with 2% FBS was used as a negative control, whereas EGM-2 CM was a positive one. Pictures of formed structures were taken after 16 hours and then analyzed with ImageJ software, with an Angiogenesis Analyzer (source: http://image.bio.methods.free.fr/ImageJ/?Angiogenesis-Analyzer-for-ImageJ&artpage=4-7#outil_sommaire_4).

### 2.5. Fibrin Bead Assay

Human aortic endothelial cells were seeded on Cytodex 3 microcarrier beads (#17-0485-03, GE Healthcare Life Sciences) and incubated for 6-8 h to allow the cells to attach. After 24 h of incubation in T25 flasks, beads with cells were resuspended in EBM-2 basal medium with 2 mg/mL fibrinogen (F3879-100MG, Sigma Aldrich), mixed with thrombin (15 *μ*L of 50 U/mL per well, T9326-150UN, Sigma Aldrich), and seeded on 24-well plates. After brief polymerization, control media or media collected from cells treated with 30 *μ*M atorvastatin were added at the top of a fibrin clot. Pictures of the beads were taken after 72 hours of incubation using a Nikon Eclipse TE 2000-U microscope.

### 2.6. Matrigel Plug Assay *In Vivo*

All procedures involving the use of animals were performed according to approved guidelines. Mice were maintained under the specific pathogen-free conditions, in individually ventilated cages, with full access to food and water. All animal experiments were approved by the Local Ethical Committee for Animal Research at the Jagiellonian University (no. 114/2014). Experiments *in vivo* were done using *Foxn1^nu^* mice. Briefly, conditioned media from PACs stimulated with 30 *μ*mol/L atorvastatin or DMSO, nonconditioned media, or wash-out media were mixed with growth factor reduced Matrigel (#356231, Corning, lot 4097006 used for all plugs) 1 : 1.5. Then, 250 *μ*L of Matrigel with media was injected subcutaneously to mice on both flanks of the abdomen. Plugs with Matrigel mixed with PBS in the same ratio served as the negative control. Additionally, PACs, stimulated with either DMSO or 30 *μ*mol/L atorvastatin for 48 hours, were also mixed with Matrigel diluted with PBS 1.5 : 1, (2.5 × 10^5^ cells in each plug) and injected subcutaneously to test the activity of cells.

Vascularization of the plug was assessed with a VEVO2100 ultrasonography system (VisualSonics) using the 3D and PowerDoppler Modes 7 days after injection. After two weeks, mice were sacrificed, and plugs were excised, then cut into small pieces and digested with the enzyme mix: 3 U/mL Liberase™ (#5410127001, Roche), 25 *μ*g/mL hyaluronidase (H4272-30MG, Sigma Aldrich), 25 *μ*g/mL DNase (#10104159001, Roche), and 3 IU/mL dispase (#17105041, Gibco) for 1 hour at 37°C. The reaction was stopped with FBS. Cells were then filtered through a 70 *μ*m cell strainer (CLS431751-50EA, Corning), centrifuged (600 g, 10 minutes), and stained in 2% FBS in PBS at 4°C for 20 minutes with the following antibodies: anti-mouse CD31 PE (#553373, clone MEC13.3, BD Biosciences, 1 *μ*g/mL), anti-mouse CD45 APC (#559864, clone 30F-11, BD Biosciences, 1 *μ*g/mL), and 0.5 *μ*g/mL DAPI (D9542-10MG, Sigma Aldrich). Stained cells were then analyzed on BD LSRFortessa. Obtained data were then analyzed with FlowJo software.

### 2.7. Multiplex Immunoassays

The concentration of factors produced by PACs was measured with the Cytokine Human 30-Plex Panel (LHC6003M, Invitrogen) on the Luminex *FLEXMAP 3D* platform according to the manufacturer's protocol.

### 2.8. NanoSight Analysis

Average size, mode size, and number of microvesicles released from PACs stimulated with DMSO or 30 *μ*mol/L atorvastatin for 48 hours were analyzed with a LM10HS microscope equipped with the LM14 488 nm laser module (Malvern Instruments Ltd., Malvern, UK) at the Department of Clinical Immunology, Institute of Pediatrics, Jagiellonian University Medical College, Kraków, Poland. Collected conditioned media were spun down at 1000 g for 10 minutes and then diluted 200x in 0.2 *μ*m-filtered PBS before the measurement.

### 2.9. Transduction of CD34^+^ Cells

To increase the efficiency of transduction, CD34^+^ cells were transduced in U-shaped 96-well plates using spinofection. Overexpression of heme oxygenase-1 was achieved with lentiviral vectors LeGO-HMOX1-iG2 encoding HMOX1 and GFP. LeGO-iG2 vector encoding GFP only was used as a control. Silencing of HMOX1 was done with lentiviral vectors pGFP-C-shLenti from OriGene. We tested 4 different sequences of shRNA and used vectors with scramble shRNA sequence as a control. Briefly, vectors were added to cells, which were subsequently spun in their culture plates for 1 hour at 800 g. 48 hours later, cells were washed and stimulated with DMSO or atorvastatin. Additionally, 10 *μ*mol/L tin protoporphyrin IX (SnPP) was used as the HMOX1 inhibitor and 10 *μ*mol/L copper protoporphyrin IX (CoPP) as the HMOX1 activator (#Sn749-9, #Co654-9, both from Frontier Scientific).

### 2.10. Gene Expression Analysis

Total RNA was isolated with phenol-chloroform extraction and reverse-transcribed with the NCode™ VILO™ miRNA cDNA synthesis kit (A11193-050, Invitrogen). The expression of genes was assessed with quantitative real-time PCR (qRT-PCR), which was performed in the StepOnePlus system (Applied Biosystems, Foster City, CA, USA) with the specific primers (*HMOX1 For:* 5′-TCTATATTTAGGGCCGCCAG-3′, *Rev:* 5′-CAGCTCCTGCAACTCCTCAAA-3′; *ACTB For:* 5′-AAGGGACTTCCTGTAACAACGCA-3′, *Rev:* 5′-CTGGAACGGTGAAGGTGACA-3′) cDNA and SYBR® Green JumpStart™ Taq ReadyMix™ (S4438-500RXN, Sigma Aldrich) according to the manufacturer's protocol.

### 2.11. Statistical Analysis

Statistical analysis of the data was performed with GraphPad Prism 7 software. Results are expressed as the mean ± SD. Data obtained in *in vitro* experiments were analyzed with a Student *t*-test when two groups of samples were used. In other cases, we used one-way or two-way ANOVA with Bonferroni *post hoc* test. The kind of statistical test applied to analyze given sets of data is provided in the description of figures.

## 3. Results

### 3.1. Obtained PBMCs Contained High Proportion of CD34^+^ HSPCs

Paracrine angiogenic activity is considered as a critical role played by myeloid angiogenic cells in the regulation of blood vessel formation. Conditioned media from such cells can be used to stimulate blood vessel growth and enhance tissue regeneration. We decided to evaluate if widely used drugs atorvastatin, acetylsalicylic acid, metformin and also sulforaphane and resveratrol are able to improve *in vitro* the proangiogenic activity of human peripheral blood CD34^+^-derived cells, which are precursors of MAC.

First, we checked the phenotype of cells that were obtained with apheresis from a healthy donor who was administered with G-CSF for 5 consecutive days. The phenotype of CD34^+^ cells was assessed within the population of single, nucleated cells from PBMC isolated from a healthy donor (Figures 1(b)–1(d)). Within Lin^−^CD45^+^ cells (Figure 1(e)), CD34^+^ cells constituted 49.4% of cells and 68.8% of them expressed CD133 (Figure 1(f)). Only a small fraction of CD45^dim^CD34^+^ (Figure 1(g)) expressed CXCR4 (Figure 1(h)), CD11b (Figure 1(i)), and CD181 (Figure 1(j)), which is a receptor for CXCL1 (IL-8), CD14, and CD16 (Figure 1(k)), and 21.9% of them expressed CD90, while CD105 was virtually absent (Figure 1(l)). The highest yield of CD34^+^ cells in culture was obtained in medium based on StemSpan-ACF, and therefore, we used it for all consecutive experiments (Supplementary Figure 2). To sum up, we obtained a large number of human CD34^+^ cells, which expressed CD133 and lacked lineage markers.

### 3.2. Media from Atorvastatin-Treated Cells Increase Tube Formation on Matrigel

Treatment of PAC with atorvastatin enhanced their paracrine angiogenic activity. HAoEC cells were treated with conditioned media harvested from cells stimulated for 48 h with atorvastatin, acetylsalicylic acid, resveratrol, sulforaphane, or metformin. Of note, conditioned media contained the stimulants as well. Therefore, to distinguish the angiogenic activity of factors released by stimulated cells from the effect of stimulants alone, we used nonconditioned media as controls. Nonconditioned media contained the same concentrations of stimulants and were incubated for the same period in the cell culture incubator as conditioned media but without cells.

**Figure 2:**
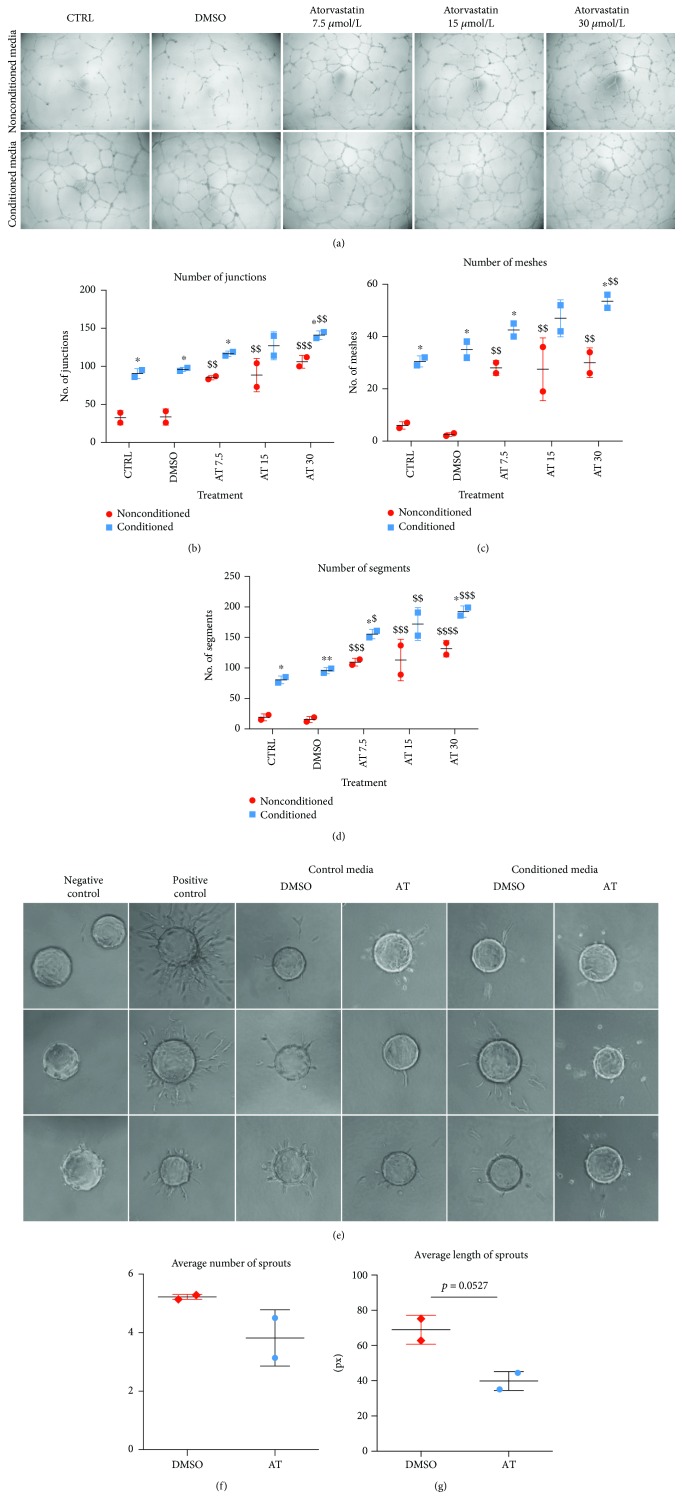
Conditioned media from PAC stimulated with atorvastatin enhance the angiogenesis in the 2D Matrigel test but not in the 3D fibrin bead assay. HAoEC cells stimulated for 16 hours with conditioned media from PAC stimulated with atorvastatin or control, nonconditioned media with atorvastatin (a); number of junctions, ^∗^ *p* < 0.05 vs. nonconditioned, ^$$^ *p* < 0.01 vs. DMSO, conditioned (b); number of meshes, ^∗^ *p* < 0.05 vs. nonconditioned, ^$$^ *p* < 0.01 vs. DMSO, conditioned (c); number of segments, ^∗^ *p* < 0.05, ^∗∗^ *p* < 0.01 vs. nonconditioned, ^$^ *p* < 0.05, ^$$^ *p* < 0.01, ^$$$^ *p* < 0.001 vs. DMSO, conditioned, two-way ANOVA with Bonferroni post hoc test, representative of 3 independent exp. (d); sprouting HAoEC cells on fibrin beads (e); an average number of sprouts (f); an average length of sprouts (g) (*N* = 2).

HAoEC cells used in the Matrigel assay *in vitro* formed tube-like structures (Figure 2(a)). First of all, conditioned media enhanced the tube formation in comparison to nonconditioned ones. However, tube-like structures formed by HAoEC cells stimulated with media from atorvastatin-treated PAC were characterized by a higher number of meshes, junctions, and segments (Figures 2(b)–2(d)). We did not observe such an effect in the case of other stimulants: resveratrol, acetylsalicylic acid, sulforaphane, or metformin (Supplementary Figure 3A-C). Interestingly, atorvastatin in nonconditioned media in all tested concentrations also improved the stability of tube-like structures formed by HAoEC cells (Figures 2(b)–2(d)). Therefore, the overall proangiogenic activity of conditioned media is rather an additive effect of both atorvastatin and factors produced by PAC.

Then, we used the fibrin bead assay, which allows assessing the formation of 3D blood vessels with a lumen (Figure 2(e)). We did not observe any differences in the number of sprouts (Figure 2(f)) or the average length of sprouts (Figure 2(g)) formed by HAoEC cells stimulated with conditioned media from atorvastatin-treated PAC in comparison to control media. Therefore, while the media from PAC stimulated with atorvastatin might have increased the proliferation of HAoEC cells, they seem not to affect the formation of the blood vessel.

### 3.3. Media from Atorvastatin-Treated CD34^+^ Cells Do Not Enhance Angiogenesis In Vivo

Then, the paracrine angiogenic activity of PAC stimulated with 30 *μ*mol/L atorvastatin, the highest dose used *in vitro* which showed a proangiogenic effect, was tested in the Matrigel plug assay *in vivo*. Similar to *in vitro* experiments, we used conditioned media from PAC treated with atorvastatin or nonconditioned media containing 30 *μ*mol/L atorvastatin and incubated in a cell culture incubator for the same period without cells. Additionally, we tested whether the paracrine angiogenic activity can be maintained in atorvastatin-treated cells after wash-out of the stimulant. Therefore, we harvested media from cells stimulated with atorvastatin for 48 hours, which were then washed and reseeded in growth medium for additional 48 hours. Media were mixed with growth factor reduced Matrigel and injected subcutaneously to *Foxn1^nu^* mice to form spherical plugs. Finally, to test the influence of the CD34^+^-derived cells themselves, we mixed atorvastatin-treated or control cells with Matrigel and injected them subcutaneously into nude mice.

**Figure 3:**
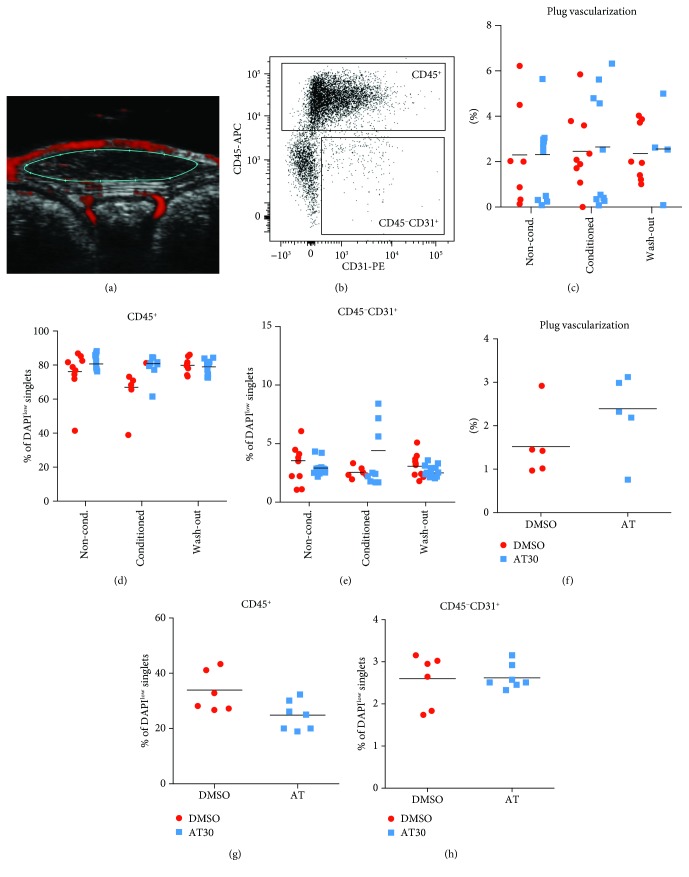
Neither conditioned media from PAC stimulated with atorvastatin nor treated cells affect the vascularization of subcutaneous Matrigel plugs. Example of an image of plug vascularization; blood flow is marked in red, borders of the plug in cyan (a). Gating for CD45^+^ cells and CD45^−^CD31^+^ cells on DAPI-negative, viable single cells (b); plug vascularization assessed with ultrasonography with the PowerDoppler mode (*N* = 10) (c); the percentage of infiltrating murine CD45^+^ cells measured with flow cytometry (d); the percentage of CD45^−^CD31^+^ endothelial cells measured with flow cytometry (e) in plugs with control nonconditioned, conditioned, or conditioned media after the wash-out of atorvastatin (*N* = 10); plug vascularization (f); the percentage of infiltrating murine CD45^+^ cells (g) or CD45^−^CD31^+^ endothelial cells (h) in plugs with PAC stimulated with 30 *μ*mol/L atorvastatin for 48 hours (*N* = 6‐7).

Angiogenic response to the media or cells was tested with 3D ultrasound imaging of blood flow in subcutaneous plugs (Figure 3(a)) and flow cytometry (Figure 3(b)). Plugs with the control, nonconditioned media, conditioned media, or conditioned media after the wash-out of atorvastatin showed similar vascularization to the control ones with vehicle only (Figures 3(b) and 3(c)). Numbers of infiltrating CD45^+^ immune cells as well as CD45^−^CD31^+^ host endothelial cells were similar in all groups (Figures 3(d) and 3(e)). Similarly, neither control PAC nor PAC stimulated with atorvastatin induced changes of plug vascularization or infiltration with CD45^+^ or CD45^−^CD34^+^ cells (Figures 3(e)–3(g)). Therefore, we concluded that the proangiogenic effect of conditioned media or PAC is too weak to be observed in the Matrigel plug assay. Furthermore, in none of the conditions tested, we have observed any changes in the local inflammation in subcutaneous plugs in mice. One has to notice, however, that the experiment was performed in immunocompromised nude mice to avoid the immune reaction to human antigens, and the conditioned media were more diluted in *in vivo* experiments than in the Matrigel assay *in vitro*.

### 3.4. Atorvastatin Does Not Change Levels of Growth Factors and Mediators of Inflammation Produced by CD34^+^ Cells

**Figure 4:**
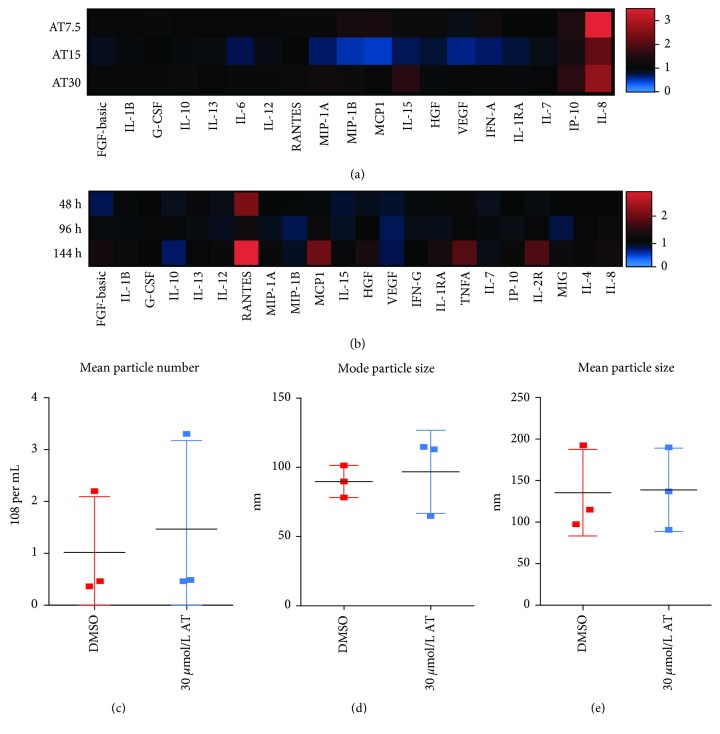
Treatment with atorvastatin does not change the levels of tested growth factors and mediators of inflammation. Heat map showing the fold change in the concentration of tested factors produced by cells treated with atorvastatin in comparison to cells treated with a vehicle (*N* = 2) (a). Heat map showing the fold change in the concentration of tested factors produced by cells treated with 30 *μ*mol/L atorvastatin for 48, 96, or 144 hours (*N* = 3) (b). Mean particle number (c); mode particle size (d); or mean particle size (e) of microvesicles produced by CD34^+^ cells treated for 48 hours either with 30 *μ*mol/L atorvastatin or with DMSO. Concentrations of secreted factors were assessed with Luminex; microvesicles were assessed with the NanoSight NTA platform (*N* = 3).

In order to further elucidate the paracrine activity of stimulated PAC, observed *in vitro*, we tested concentration of 30 human growth factors and mediators of inflammation in conditioned media from PAC stimulated with 7.5, 15, or 30 *μ*mol/L atorvastatin. Only IL-8 seemed to increase upon stimulation with atorvastatin (Figure 4(a), Supplementary Figure 4). However, it was not reproduced in further experiments, even when cells were stimulated for a more extended period, up to 144 hours (Figure 4(b), Supplementary Figure 5). Since microvesicles may mediate the proangiogenic effect of circulating cells, we assessed the mean particle number, mode, and mean particle sizes using the nanoparticle tracking analysis. None of the parameters tested was affected by atorvastatin (Figures 4(c)–4(e)).

### 3.5. Atorvastatin Does Not Affect the *HMOX1* Expression in CD34^+^ Cells

**Figure 5:**
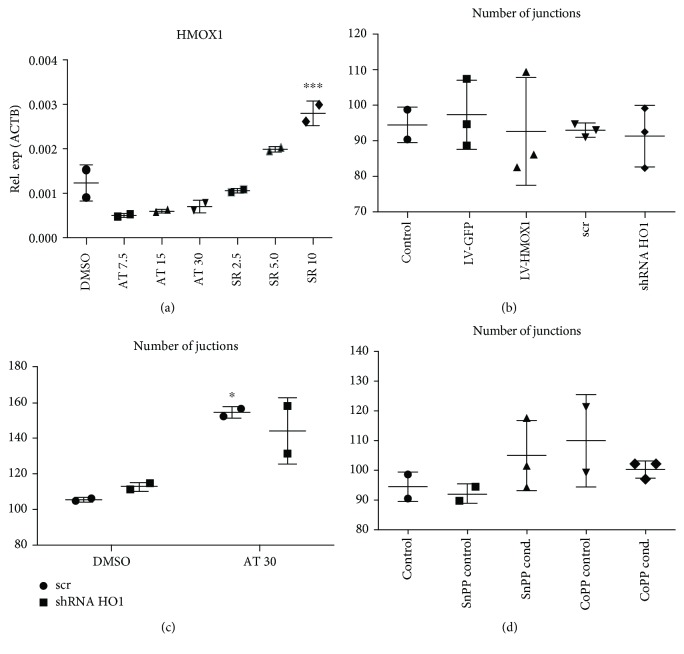
Atorvastatin does not stimulate *HMOX1* expression. 10 *μ*mol/L sulforaphane induces higher levels of *HMOX1* gene expression after 6 hours of treatment; ^∗∗∗^ *p* < 0.001, one-way ANOVA with Bonferroni post hoc test, *N* = 2 (a). Neither *HMOX1* overexpression, nor silencing, nor modulation of *HMOX1* activity affects the paracrine angiogenic activity of PAC. Number of junctions in the Matrigel test in HAoEC cells stimulated with media from PAC transduced with lentiviral vectors encoding for either *HMOX1* or *shRNA HMOX1*, *N* = 3 (b); number of junctions in the Matrigel test in HAoEC cells stimulated with media from PAC with silenced *HMOX1* stimulated with atorvastatin; ^∗^ *p* < 0.05 vs. control cells treated with shSCR and DMSO, *N* = 2 (c); number of junctions in the Matrigel test in HAoEC cells stimulated with control, nonconditioned media, or conditioned media from PAC stimulated with 10 *μ*mol/L tin or cobalt protoporphyrin IX (SnPP or CoPP, respectively), *N* = 3 (d).

Finally, we assessed whether stimulation with atorvastatin could affect the expression of heme oxygenase-1, a known proangiogenic factor, which was crucial for the paracrine angiogenic activity of mouse PAC cells [27]. The only stimulant that enhanced *HMOX1* expression in PAC was sulforaphane, a known inductor of Nrf2 transcription factor activity (Figure 5(a)). However, sulforaphane did not affect the paracrine angiogenic activity of the tested population of PAC (Supplementary Figure 3). On the other hand, atorvastatin, which increased PAC paracrine angiogenic activity, did not change *HMOX1* transcript levels (Figure 5(a)). Furthermore, neither overexpression of *HMOX1* with lentiviral vectors nor *HMOX1* silencing with shRNA affected the paracrine proangiogenic activity of PAC (Figure 5(b)). HAoECs treated with media from PAC with silenced *HMOX1* and stimulated with atorvastatin formed a similar number of junctions (Figure 5(c)). Last but not least, neither chemical activation nor inhibition of HMOX1 activity affected the angiogenic activity of media from PAC (Figure 5(d)). Therefore, we conclude that in PAC, *HMOX1* is not vital for the regulation of their paracrine proangiogenic activity.

## 4. Discussion

The main finding of the study is that conditioned media from human peripheral blood PAC-stimulated and atorvastatin have additive proangiogenic activity *in vitro* in the Matrigel assay but not *in vivo* in the subcutaneous Matrigel plug model. The effect *in vitro* was not mediated by heme oxygenase-1 since the *HMOX1* expression level was not affected by atorvastatin. On the other hand, increased expression of *HMOX1* induced in sulforaphane-treated cells did not lead to any changes in paracrine proangiogenic activity. Interestingly, conditioned media from C2C12 myoblast cells overexpressing heme oxygenase-1 had higher proangiogenic activity *in vivo* in ischemic hind limbs of *db/db* diabetic mice [29]. Media from the latter cells were devoid of antiangiogenic PEDF and BGN, which were present in media from control cells, and contained more proangiogenic PPlase A, MIF, haptoglobin, and galectin-1 [29].

Atorvastatin alone in nonconditioned media enhanced tube formation in the Matrigel assay. However, the impact of atorvastatin on endothelial cells and their function is controversial. High concentrations of atorvastatin were previously shown to inhibit HUVEC sprouting and growth factor-induced proliferation *in vitro* [30]. In our experiments, we used aortic endothelial cells and culture media with reduced concentration of serum or even serum-free media. Furthermore, we stimulated HAoEC with atorvastatin only for a short period, sufficient to form tube-like structures on Matrigel, while Dulak et al. stimulated cells for 48 hours [30]. Interestingly, 1 *μ*mol/L atorvastatin enhanced *VEGF* and *IL8* promoter activities but decreased their production by human microvascular endothelial cells in hypoxia [31]. Furthermore, Dang and coworkers showed that 10 *μ*mol/L atorvastatin did not affect tube formation by HUVEC in an overnight stimulation [32]. Atorvastatin also decreased endothelial senescence and dysfunction induced by angiotensin II [32]. On the other hand, atorvastatin administered orally inhibited inflammatory angiogenesis and production of VEGF in sponge transplants in mice [33]. When administered in low dose (10 mg/kg/day, orally) but not high dose (30 mg/kg/day), atorvastatin improved angiogenesis in ischemic limbs in the murine hind limb ischemia model [34]. It also enhanced the expression of proangiogenic VEGF, IL-8, and Hmox1 [34].

In HUVEC, atorvastatin increased heme oxygenase-1 mRNA but did not influence HMOX1 protein levels [30]. Levels of *HMOX1* were also unaffected in human microvascular endothelial cells (HMEC1) treated with a clinically relevant concentration of atorvastatin (0.1 *μ*mol/L) [35]. In our hands, we did not observe significant changes in *HMOX1* expression in PAC stimulated with 30 *μ*mol/L atorvastatin.

*In vivo*, in mouse lungs and hearts, atorvastatin (and other statins) increased heme oxygenases' activity and heme oxygenase-1 but not 2 mRNA and protein levels [36]. In other experimental settings, high doses of simvastatin and lovastatin were able to increase heme oxygenase-1 levels in HUVEC independently of NF*κ*B and Nrf2 signalling [37]. Furthermore, *Hmox1* was also upregulated by statins in RAW264.7 macrophages, and p38 MAPK, ERK, and protein kinase G mediated its induction [23]. Therefore, we concluded that the impact of atorvastatin is most probably dependent on the cell type.

Enhanced angiogenic properties of atorvastatin-stimulated cells observed *in vitro* in the Matrigel tube formation assay were not confirmed with the fibrin bead assay *in vitro* and *in vivo* Matrigel plug assay. However, in both tests, the conditioned media were more diluted than in the Matrigel tube formation assay. Interestingly, there was no difference in the number of CD31^+^ cells in Matrigel plugs implanted both peritoneally and subcutaneously in rats administered orally with 15 mg/kg/day of atorvastatin for seven days [38]. However, the number of *α*SMA^+^ smooth muscle cells was decreased in subcutaneous implants, which may suggest the impact of atorvastatin on stability of newly formed blood vessels [38].

Matrigel plugs with conditioned media were injected subcutaneously to *Foxn1^nu^* mice, which are not able to produce T lymphocytes, but have relatively normal innate immunity. One may speculate that the effect of media on angiogenesis could be hampered by infiltrating murine immune cells, which comprised the majority of cells found in plugs regardless of the type of media used (Figure 3(d)). Importantly, conditioned media from CD34^+^-derived cells stimulated with all compounds contained similar levels of growth factors and inflammatory mediators. However, to fully address the issue of the immune response to human proteins or cells in the proposed experimental setting, we would have to use humanised animals.

Finally, prolonged stimulation with atorvastatin tended to increase levels of MCP-1, which can induce arteriogenesis [39, 40]. However, in our study, we did not observe such an effect *in vivo* with ultrasound measurement of blood flow inside the plugs. Moreover, atorvastatin was shown previously to inhibit MCP-1 production in rabbit vascular smooth muscle cells [41] and U937 monocytic cell line [42] and possibly affecting the final outcome *in vivo*.

To conclude, the effects of atorvastatin on the paracrine angiogenic activity of CD34^+^-derived cells could be observed only *in vitro* and not *in vivo*. Levels of tested cytokines and tested parameters of microvesicles in media that enhanced angiogenesis were not changed. Therefore, we believe that the effect observed *in vitro* most probably results rather from the orchestrated small changes in many factors than a single one.

## Data Availability

The data used to support the findings of this study are available from the corresponding author upon request.

## Supplementary Materials

Supplementary Materials Supplementary Table 1: composition of media tested for the culture of human peripheral blood CD34^+^. Supplementary Figure 1: gating controls for the phenotype analysis of CD34^+^ cells. Supplementary Figure 2: flow cytometry analysis of nonadherent cells cultured in tested media. Supplementary Figure 3: the effects of acetylsalicylic acid, resveratrol, sulforaphane, and metformin on paracrine angiogenic activity of PAC. Supplementary Figure 4: production of growth factors or mediators of inflammation after the stimulation with 30 *μ*M atorvastatin. Supplementary Figure 5: prolonged treatment with 30 *μ*M atorvastatin has no or low effect on the levels of mediators of inflammations or growth factors produced by PAC.
