# Supplementary material for: Atorvastatin and Conditioned Media from Atorvastatin-Treated Human Hematopoietic Stem/Progenitor-Derived Cells Show Proangiogenic Activity In Vitro but Not In Vivo
Source: Mediators Inflamm. 2019 Jul 16;2019:1868170. doi: 10.1155/2019/1868170 (PMC6664685; doi:10.1155/2019/1868170)
Supplement: Supplementary Materials — Supplementary Table 1: composition of media tested for the culture of human peripheral blood CD34+. Supplementary Figure 1: gating controls for the phenotype analysis of CD34+ cells. Supplementary Figure 2: flow cytometry analysis of nonadherent cells cultured in tested media. Supplementary Figure 3: the effects of acetylsalicylic acid, resveratrol, sulforaphane, and metformin on paracrine angiogenic activity of PAC. Supplementary Figure 4: production of growth factors or mediators of inflammation after the stimulation with 30 μM atorvastatin. Supplementary Figure 5: prolonged treatment with 30 μM atorvastatin has no or low effect on the levels of mediators of inflammations or growth factors produced by PAC. [file 1868170.f1.pdf]

Supplementary Table 1 *Composition of media tested for the culture of human peripheral blood CD34+. Cells were cultured on cell culture dishes coated with either CellStart CTS (A1014201, Gibco) or 20 µg/ml human plasma fibronectin (F2006, Sigma Aldrich).*

Medium	Supplement
StemPro MSC SFM Xenofree (A1067501, Gibco)	-
MesenCult-XF (#05420, Stem Cell Technologies)	-
StemSpan-ACF (#09855, Stem Cell Technologies	rhSCF (AF-300-07, Peprotech) 100 ng/ml, rhFlt3-L (AF-300-19, Peprotech) 100 ng/ml, rhTPO (AF-300-18, Peprotech) 20 ng/ml, rhVEGF (AF-100-20, Peprotech) 50 ng/ml, rhIL-6 (AF-200-06, Peprotech) 20 ng/ml
EBM-2 (CC-3156, Lonza)	rhSCF 100 ng/ml, rhFlt3-L 100 ng/ml, rhTPO 20 ng/ml, rhVEGF 50 ng/ml, rhIL-6 20 ng/ml
EBM-2	KnockOut Serum Replacement XenoFree CTS (A3181502, Gibco), rhSCF 100 ng/ml, rhFlt3-L 100 ng/ml, rhTPO 20 ng/ml, rhVEGF 50 ng/ml, rhIL-6 20 ng/ml
EBM-2	KnockOut Serum Replacement XenoFree CTS, 1% Pen/Strep, 0.04% hydrocortisone, 0.1% heparin, 0.1% ascorbic acid, IGF-1 50 ng/ml, EGF 10 ng/ml, FGF-2 50 ng/ml, VEGF 50 ng/ml (all from bullet kit CC-4176, Lonza)
EBM-2	ITS+ (#354352, BD Biosciences), 1% Pen/Strep, 0.04% hydrocortisone, 0.1% heparin, 0.1% ascorbic acid, IGF-1 50 ng/ml, EGF 10 ng/ml, FGF-2 50 ng/ml, VEGF 50 ng/ml
EBM-2	ITS+, rhSCF 100 ng/ml, rhFlt3-L 100 ng/ml, rhTPO 20 ng/ml, rhVEGF 50 ng/ml, rhIL-6 20 ng/ml

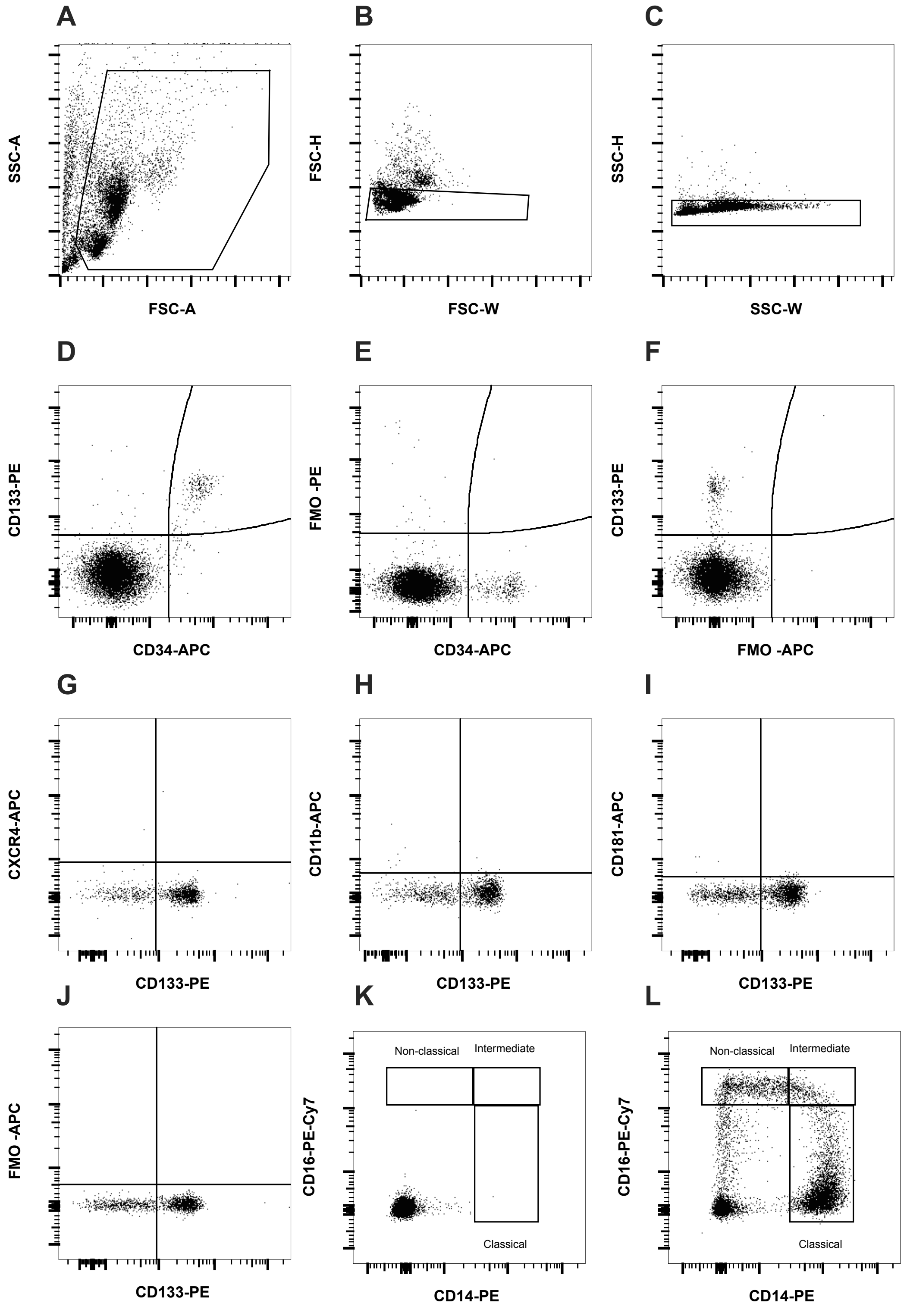

Supplementary Figure 1. Gating controls for the phenotype analysis of CD34^{+} cells. Cells (**A**), singlets gated on FSC-H vs FSC-W (**B**), singlets gated on SSC-H vs SSC-W (**C**), staining for CD133-PE and CD34-APC (**D**), fluorescence minus one (FMO) control for CD133-PE (**E**), FMO control for CD34-APC (**F**), staining for CD133-PE and CXCR4-APC on CD34+ cells (**G**), staining for CD133-PE and CD11b-APC on CD34+ cells (**H**), staining for CD133-PE and CD181-APC on CD34+ cells (**I**), FMO control for CXCR4-APC, CD11b-APC, CD181-APC (**J**), gating for CD16-PE-Cy7 and CD14-PE on CD34+ cells (**K**), staining for CD16-PE-Cy7 and CD14-PE on CD34- cells (**L**)

A

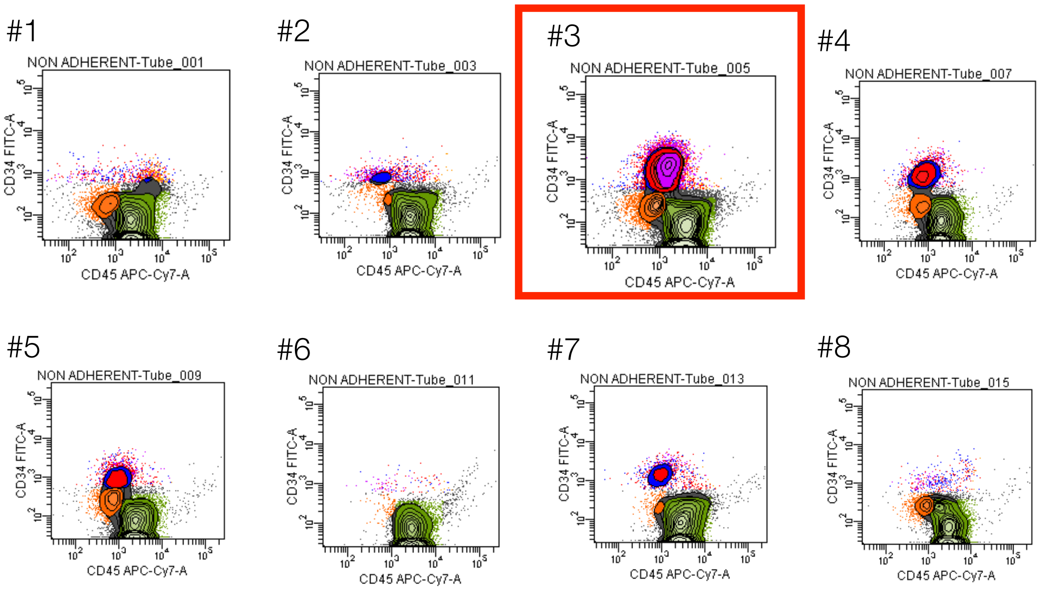

B

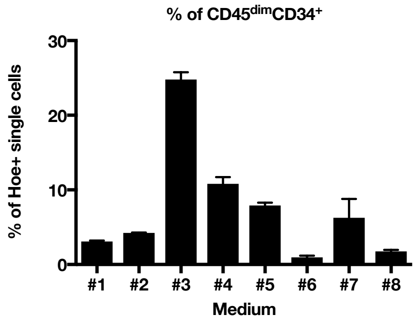

Supplementary Figure 2. Flow cytometry analysis of non-adherent cells cultured in tested media (**A**) percentage of CD45^{dim}CD34^{+} cells in tested media after 7 days of culture.

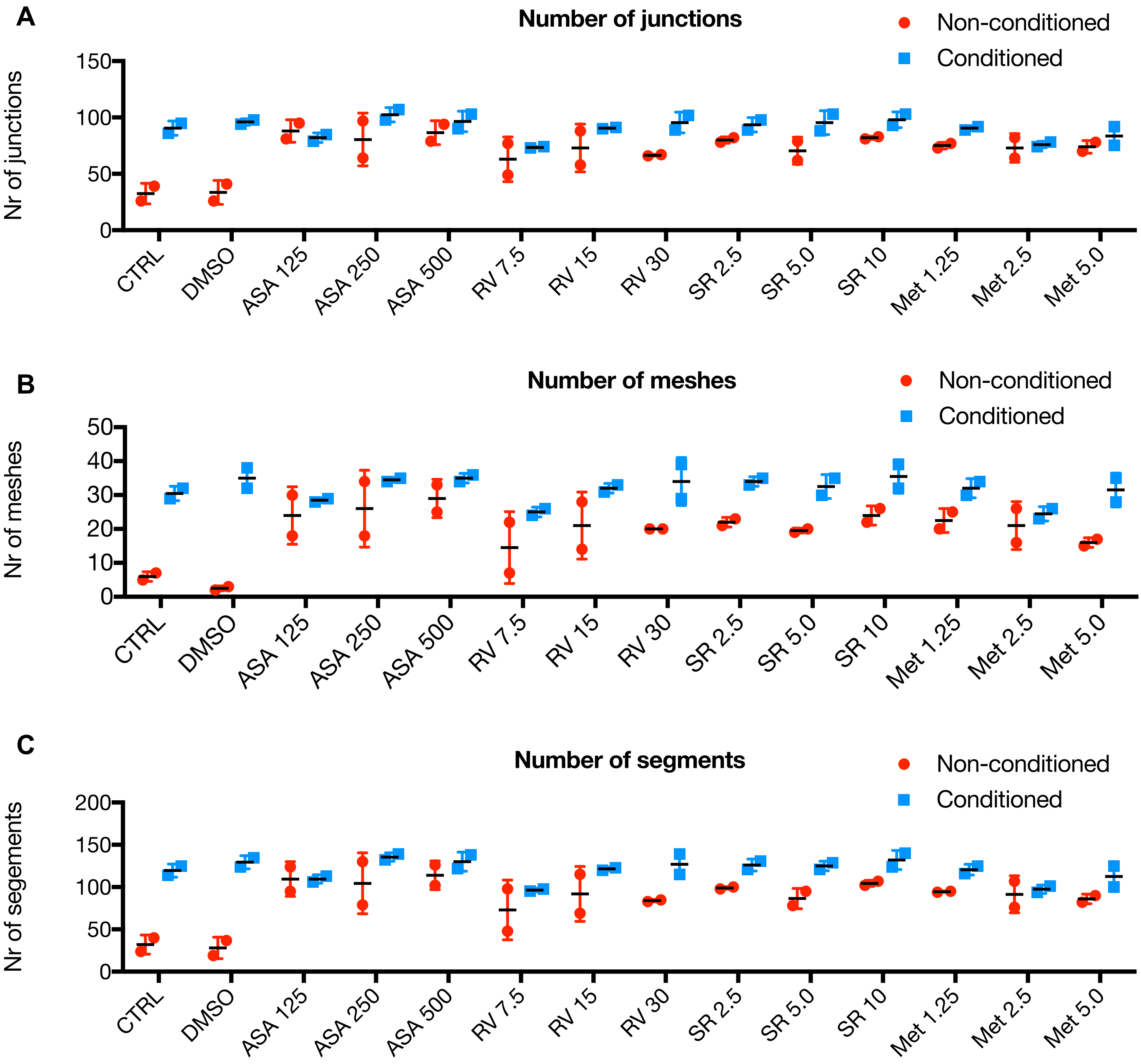

Supplementary Figure 3. The effects of other stimulants on paracrine angiogenic activity of PAC: 125, 250 or 500 µM acetylsalicylic acid (ASA), 7.5, 15 or 30 µM resveratrol (RV), 2.5, 5.0 or 10 µM sulforaphane (SR) or 1.25, 2.5 or 5.0 mM metformin (Met) did not affect the paracrine proangiogenic activity of PAC *in vitro*. The number of junctions (**A**), meshes (**B**), and segments (**C**) in HAoEC seeded on Matrigel and stimulated with control, non-conditioned or conditioned media from PAC stimulated with abovementioned concentrations of stimulants. The figure shows the representative graphs from 3 experiments.

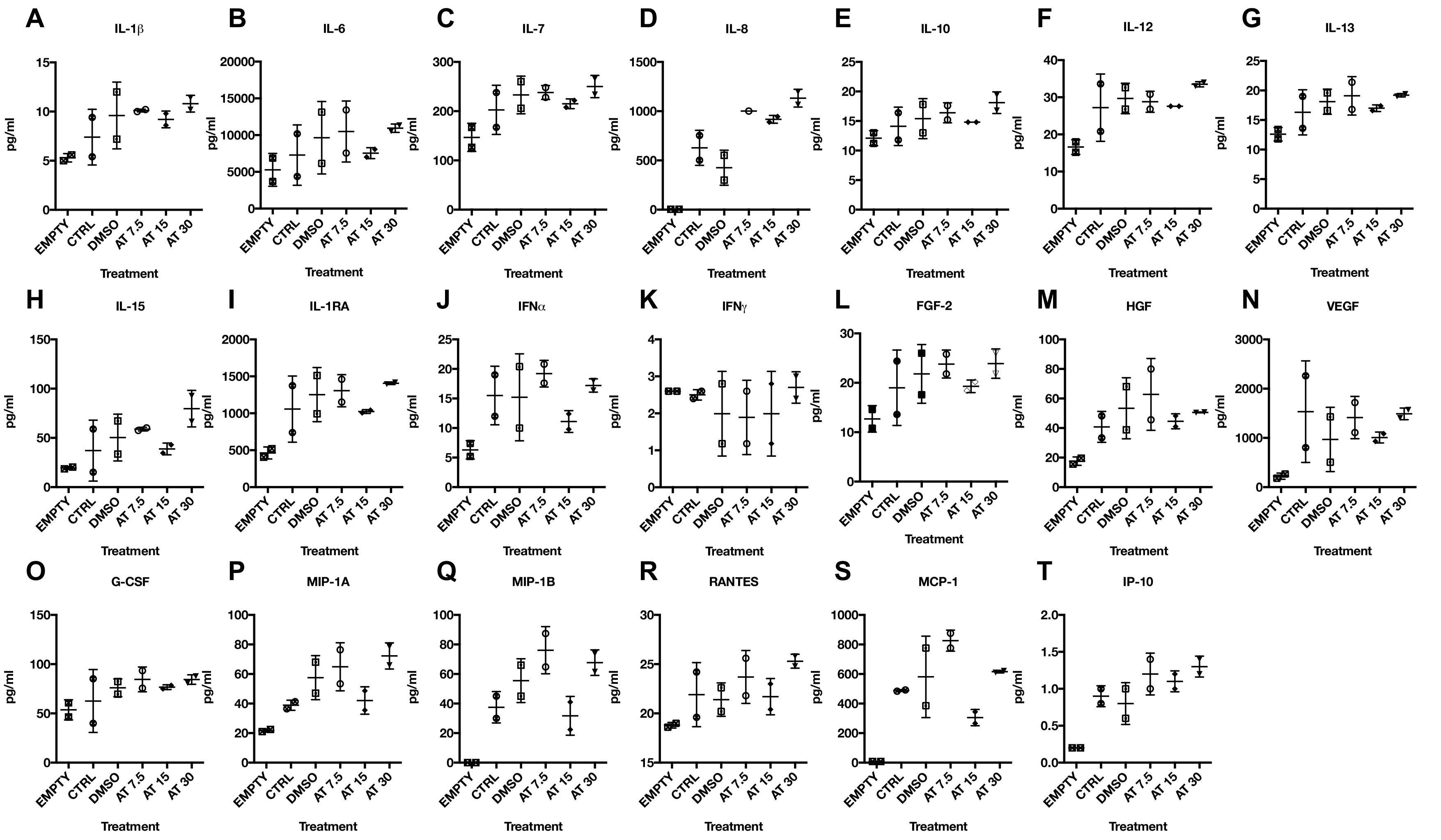

Supplementary Figure 4. Treatment with 30 μM atorvastatin does not affect the production of tested growth factors or mediators of inflammation. Concentrations of IL-1β (**A**), IL-6 (**B**), IL-7 (**C**), IL-8 (**D**), IL-10 (**E**), IL-12 (**F**), IL-13 (**G**), IL-15 (**H**), IL-1RA (**I**), IFNα (**J**), IFNγ (**K**), FGF-2 (**L**), HGF (**M**), VEGF (**N**), G-CSF (**O**), MIP-1A (**P**), MIP-1B (**R**), RANTES (CCL5) (**R**), MCP-1 (**S**), IP-10 (**T**) in conditioned media from PAC stimulated with either vehicle or atorvastatin. N=2

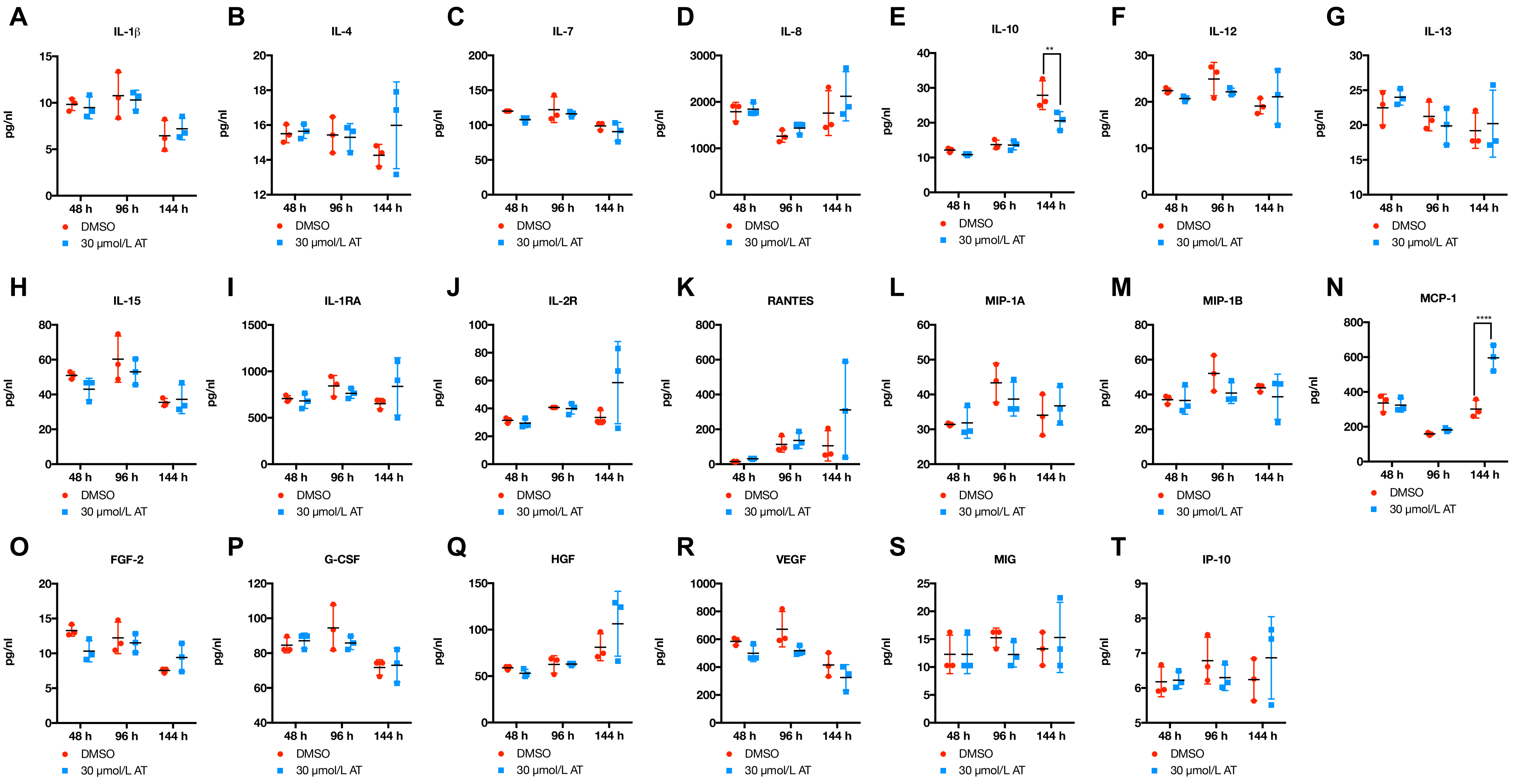

Supplementary Figure 5. Prolonged treatment with 30 µM atorvastatin has no or low effect on the levels of mediators of inflammations or growth factors produced by PAC. Concentrations of IL-1β (**A**), IL-4 (**B**), IL-7 (**C**), IL-8 (**D**), IL-10 (**E**), IL-12 (**F**), IL-13 (**G**), IL-15 (**H**), IL-1RA (**I**), IL-2R (**J**), RANTES (**K**), MIP-1A (**L**), MIP-1B (**M**), MCP-1 (**N**), FGF-2 (**O**), G-CSF (**P**), HGF (**R**), VEGF (**R**), MIG (**S**), IP-10 (**T**) in conditioned media from PAC stimulated with either vehicle or atorvastatin for 48, 96, or 144 hours, N=3.
